# Regulation of Myosin-5b by Rab11a and the Rab11 family interacting protein 2

**DOI:** 10.1042/BSR20181252

**Published:** 2019-01-08

**Authors:** Huan-Hong Ji, Lin-Lin Yao, Chang Liu, Xiang-dong Li

**Affiliations:** 1 Group of Cell Motility and Muscle Contraction, State Key Laboratory of Integrated Management of Pest Insects and Rodents, Institute of Zoology, Chinese Academy of Sciences, Beijing 100101, China; 2 University of Chinese Academy of Sciences, Beijing 100049, China

**Keywords:** allosteric regulation, actin, endosome, myosins, molecular motors

## Abstract

Mammalian myosin-5b (Myo5b) plays a critical role in the recycling of endosomes to the plasma membrane via the interactions with Rab11a and the Rab11 family interacting protein 2 (FIP2). However, it remains unclear on how Rab11a and FIP2 are coordinated in tethering Myo5b with the vesicles and activating the motor function of Myo5b. In the present study, we show that Rab11a binds to the globular tail domain (GTD) of Myo5b and this binding abolishes the head–GTD interaction of Myo5b, thus activating the motor function of Myo5b. On the other hand, FIP2 directly interacts with both Rab11a and the tail of Myo5b, and the binding of FIP2 to Myo5b does not affect Myo5b motor function. Moreover, Rab11a displays higher affinity to FIP2 than to Myo5b, suggesting that Rab11a binds preferentially to FIP2 than to Myo5b. Based on the current findings, we propose that the association of Myo5b with vesicles is mediated by FIP2, which bridges Myo5b and the membrane-bound Rab11a, whereas the motor function of Myo5b is regulated by Rab11a.

## Introduction

Mammals have three class V myosin genes, encoding Myo5a, Myo5b and Myo5c [1]. Myo5b plays a critical role in the recycling of endosomes to the plasma membrane, and this process is mediated by two Myo5b-binding proteins, i.e. Rab11a and the Rab11 family interacting protein 2 (hereafter abbreviated as FIP2) [2,3]. Myo5b, Rab11a and FIP2 are supposed to form a ternary complex, thus enabling actin-filament-dependent transport of recycling endosomes [2,3]. However, it remains unclear on how those three proteins are coordinated in the recycling of endosomes.

Myo5b consists of two identical heavy chains that dimerize through the coiled-coil regions to form a homodimer. At the amino terminus of Myo5b is the motor domain (also called the head), followed by the lever arm (also called neck region). The remaining residues form the tail, comprising the proximal tail that contains a series of coiled-coils and the distal globular tail domain (GTD). The tail mediates the association of Myo5b with specific membrane-bound organelles via direct or indirect interaction with Rab proteins [4–6]. In addition, the GTD of Myo5b functions as the inhibitory domain, which binds to the motor domain and inhibits its motor function [3].

The Rab family of small G proteins comprises more than 60 members, which regulate various aspects of membrane dynamics, such as vesicle motility, docking and fusion [7]. Rabs are anchored to the vesicles via the C-terminal prenylation at cysteine residues, and function as molecular switches in recruiting various effector proteins. Several Rabs have been shown to interact directly or indirectly with class V myosin. For instance, Rab8a, Rab10 and Rab11a directly bind to the tail of Myo5a and Myo5b [8–10], and Rab27a associates with Myo5a via melanophilin [11,12]. The Rab11 subfamily, comprising Rab11a, Rab11b and Rab25 (also known as Rab11c), regulate the recycling of endosomes via the interactions with Myo5b and FIP2 [13].

FIP2 contains three distinct domains, i.e., the N-terminal phospholipid-binding C2 domain that binds preferentially to membrane lipids, the central Myo5b binding domain (MBD) and the C-terminal Rab11-binding domain (RBD), which interacts with all three members of Rab11 family [14]. In the crystal structure of FIP2–RBD/Rab11a complex, FIP2–RBD forms a central α-helical coiled-coil, with both helices binding to Rab11a, thus forming a Rab11a–(FIP2–RBD)_2_–Rab11a heterotetramer [15]. Interestingly, Rab11a uses same surface to interact with Myo5b–GTD and FIP2–RBD [16]. Therefore, it was proposed that FIP2–RBD might compete with Myo5b–GTD in binding to Rab11a [15,16]. In addition to directly interacting with the GTD of Myo5b, Rab11a may associate with Myo5b via FIP2.

Because the GTD serves as both the inhibitory domain and the binding site for adaptor proteins, it is expected that adaptor proteins will interfere the head–GTD interaction, thus relieving the inhibition on the motor domain [17–19]. Consistent with this notion, we recently found that Drosophila Rab11 interacts with the GTD of Drosophila myosin-5 and activates its motor function [20]. It is of interest to know whether the motor function of mammalian Myo5b is regulated by Rab11a and FIP2.

In the present study, we demonstrate that Rab11a binds to Myo5b–GTD and abolishes the head–GTD interaction of Myo5b, thus activating Myo5b motor function. On the other hand, we show that FIP2 tethers Rab11a and Myo5b without altering Myo5b motor function. We propose that the association of Myo5b with the vesicle is mediated by FIP2 and Rab11a, and the motor function of Myo5b is activated by Rab11a.

## Materials and methods

### Materials

AccuScript Reverse Transcriptase and PfuUltra II Fusion HS DNA Polymerase were purchased from Stratagene (La Jolla, CA). Restriction enzymes and modifying enzymes were from New England BioLabs (Beverly, MA). Anti-Flag M2 antibody, anti-Flag M2 affinity agrose, phosphoenolpyruvate (PEP), 2,4-dinitrophenyl-hydrazine, pyruvate kinase and glutathione (GSH) were from Sigma-Aldrich (St. Louis, MO). Flag peptide (DYKDDDDK) was synthesized by Augct (Beijing, China). Oligonucleotides were synthesized by Invitrogen (Beijing, China). Glutathione Sepharose 4 Fast Flow (GSH-Sepharose) was from GE Healthcare. EDTA-free Protease Inhibitor Cocktail was from Roche. Actin and calmodulin (CaM) were prepared as described previously [20].

### Expression and purification of Myo5b constructs

**Figure 1:**
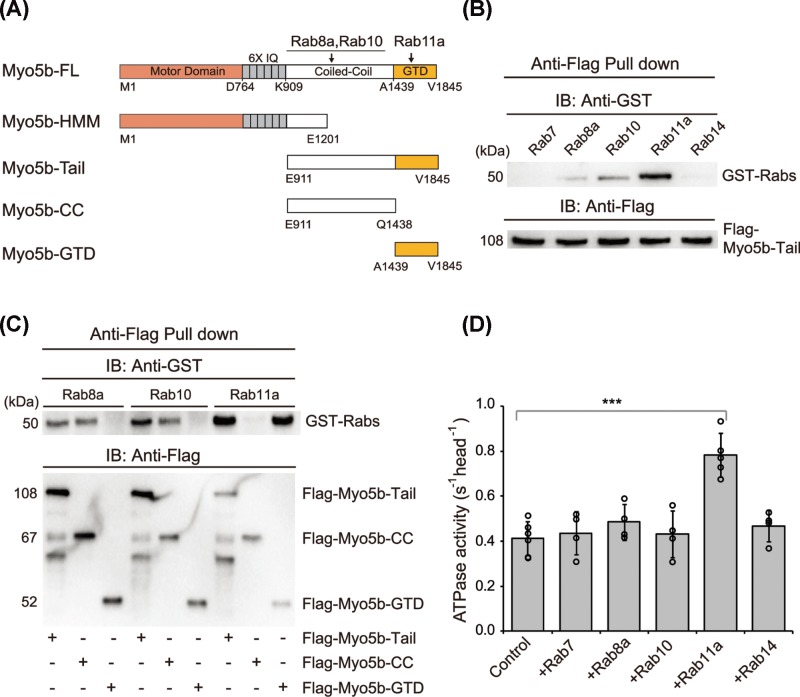
Effects of Rabs on Myo5b ATPase activity (**A**) Schematic primary structure of Myo5b and its binding sites with Rabs. Myo5b-FL, the full-length Myo5b; IQ, the CaM binding site; GTD, the C-terminal globular tail domain. (**B**) Anti-Flag pull down assays reveal the interaction of Myo5b tail with GST–Rab8a, 10 and 11a. (**C**) Anti-Flag pull down of Flag-Myo5b tail constructs with GST-Rabs. (**D**) Effects of Rabs on Myo5b ATPase activity. The ATPase activity was measured in the presence of ∼0.1 μM Myo5b-FL, 40 μM actin and 12 μM GST-Rab in a solution containing 20 mM MOPS-KOH (pH 7.0), 200 mM KCl, 1 mM MgCl_2_, 1 mM DTT, 0.25 mg/ml BSA, 12 μM CaM, 0.5 mM ATP, 2.5 mM PEP, 20 U/ml pyruvate kinase and 1 mM EGTA. Control shows the ATPase activity of Myo5b-FL in the absence of GST-Rab. Values are mean ± S.D. from at least four independent assays; ***P<0.001. Anti-Flag pull down assays were repeated three times and typical results are presented.

Full-length and heavy meromyosin (HMM) fragments of rat Myo5b were expressed in sf9 insect cells using baculovirus expression system and purified as described previously [3]. To prepare Myo5b tail constructs (Myo5b–Tail, Myo5b–CC and Myo5b–GTD, Figure 1A), their cDNAs were amplified by PCR and subcloned into either pET30a-Flag (a modified pET30a vector encoding an N-terminal Flag-tag) or pGEX4T1 vector (encoding an N-terminal GST tag) using EcoRI and XhoI sites. These constructs were expressed in Escherichia coli BL21(DE3) and purified by anti-Flag M2 affinity chromatography or GSH-Sepharose according to standard procedures. The purified proteins were dialyzed against 10 mM Tris-HCl (pH 7.5), 0.2 M NaCl and 1 mM DTT at 4°C overnight. The concentrations of the purified proteins were determined based on the absorbance at 280 nm.

### Expression and purification of Rabs and FIP2

The cDNAs of FIP2 were amplified by RT-PCR with human brain cDNA as template. The cDNAs of full-length FIP2 and the truncated constructs FIP2-ΔRBD (amino acids 1–476) and FIP2–RBD (amino acids 452–512) were amplified by PCR and subcloned into pET30a-Flag, a modified pET30a encoding an N-terminal Flag sequence.

The cDNAs of Rabs were amplified by RT-PCR with human kidney cDNA (for Rab7, Rab8a, Rab10 and Rab14) or human brain cDNA (for Rab11a) as template and subcloned into pGEX4T1 vector. The expression and purification of Rabs and FIP2 proteins were similar to those of Myo5b–tail. The concentrations of proteins were measured by absorbance at 280 nm. Prior to function assays, Rab proteins were incubated with a 5-fold excess of GTPγS (Millipore, MA) and MgCl_2_ for 30 min at 25°C to obtain the active form of Rabs (GTP-bound GST–Rabs).

### ATPase assay

The ATPase activity of Myo5b was measured in a plate-based, ATP regeneration system as described previously with slight modification [21]. Unless otherwise indicated, Myo5b ATPase activity was measured at 25°C in 20 mM MOPS-KOH (pH7.0), 200 mM KCl, 1 mM MgCl_2_, 1 mM DTT, 0.25 mg/ml BSA, 12 μM CaM, 0.5 mM ATP, 2.5 mM PEP, 20 U/ml pyruvate kinase, ∼100 nM Myo5b-FL or ∼40 nm Myo5b–HMM, 40 μM actin, 1 mM EGTA and indicated concentrations of other proteins, such as GST–Rab11a, FIP2–RBD, GST–GTD etc.

### Anti-Flag pull down assay

Anti-Flag pull down of Myo5b-tail constructs with Rabs was performed as follows. One hundred microliters of 1 μM Flag-Myo5b-tail and 1.6 μM GST-Rabs in Buffer-A [20 mM MOPS-KOH (pH 7.0), 1 mM MgCl_2_, 1 mM EGTA and 1 mM DTT] containing 100 mM NaCl and 3 μM GTPγS were mixed with 15 μl anti-Flag agarose and incubated with rotation at 4°C for 1 h. The beads were collected by brief centrifugation and then washed with 100 μl of Buffer A containing 100 mM NaCl and 1 μM GTPγS for three times. The bound proteins were eluted with 40 μl of 0.1 mg/ml Flag in Buffer A. The eluted protein were separated by SDS−PAGE (4–20%) and visualized by Western blot using Anti-Flag or Anti-GST antibody.

Anti-Flag pull down of Flag-Myo5b–HMM with GST–GTD and GST–Rab11a was performed as follows. Eighty microliters of 0.8 μM Flag-Myo5b-HMM, 0.8 μM GST-Myo5b-GTD, 0–20 μM GST–Rab11a and 10 μM CaM in Buffer-A containing 150 mM NaCl and 20 μM GTPγS were mixed with 20 μl of anti-Flag agarose and incubated with rotation at 4°C for 1 h. The beads were collected by brief centrifugation and then washed with 100 μl of Buffer-A containing 50 mM NaCl and 10 μM GTPγS for four times. The bound proteins were eluted with 50 μl of 0.1 mg/ml Flag in Buffer-A containing 50 mM NaCl and 10 μM GTPγS. The eluted protein were separated by SDS−PAGE (4–20%) and visualized by Coomassie Brilliant Blue staining. Note: We found that washing the anti-Flag beads for four times was optimal for showing the effects of Rab11a on the binding of Myo5b–HMM with GST–GTD, although it was not sufficient to complete remove Rab11a. More extensive washing was able to completely remove Rab11a, but the pulled down samples would contain too little amount of GST–GTD to be detected by Coomassie Brilliant Blue staining.

### GST pull down assay

GST pull down of GST–Rab11a with Myo5b–GTD and FIP2–RBD was performed as follows. One hundred microliters of 0.5 μM GST–Rab11a, 0–1.0 μM Flag-Myo5b-GTD and 0–1.0 μM Flag-FIP2-RBD in Buffer-B [20 mM MOPS-KOH (pH 7.0), 1 mM MgCl_2_, 1 mM EGTA, 1 mM DTT and protease inhibitor cocktail (Roch)] containing 100 mM NaCl and 3 μM GTPγS were mixed with 20 μl of GSH-Sepharose and incubated with rotation at 4°C for 1 h. The bead was collected by brief centrifugation and washed with 100 μl of Buffer-B containing 50 mM NaCl and 1 μM GTPγS for five times. The bound proteins were eluted with 50 μl of 20 mM GSH in Tris-HCl (pH 8.0), 100 mM NaCl and 1mM DTT. The eluted proteins were separated by SDS−PAGE (4–20%) and visualized Western blot.

GST pull down of GST–Rab11a with Myo5b–Tail and FIP2 or FIP2–RBD was performed as follows. One hundred microliters of 0.5 μM GST–Rab11a, 0–0.5 μM Flag-Myo5b-Tail and 0–1.0 μM Flag-FIP2 or Flag-FIP2-RBD in Buffer-B containing 100 mM NaCl and 3 μM GTPγS were mixed with 20 μl of GSH-Sepharose and incubated with rotation at 4°C for 1 h. The bead was collected by brief centrifugation and washed with 100 μl of Buffer-B containing 50 mM NaCl and 1 μM GTPγS for five times. The bound proteins were eluted with 50 μl of 20 mM GSH in Tris-HCl (pH 8.0), 100 mM NaCl and 1 mM DTT. The eluted proteins were separated by SDS−PAGE (4–20%) and visualized by Coomassie Brilliant Blue staining.

## Results

### Rab11a specifically binds to the GTD of Myo5b and activates the motor function of Myo5b

A number of Rab G proteins, including Rab8a, Rab10, Rab11a and Rab14, have been shown to directly interact with class V myosin [8,9,22]. To determine their interaction with Myo5b, we performed pull down assay using purified GST-tagged Rab proteins and Flag-tagged Myo5b proteins. GST-tagged Rab proteins were expressed in *Escherichia coli* and purified by GSH-Sepharose chromatography. Flag-tagged Myo5b proteins, including the full-length and the truncated constructs (Figure 1A), were expressed in Sf9 cells or *E. coli* and purified by Anti-Flag affinity chromatography. We found that Flag-Myo5b-tail specifically pulled down Rab8a, 10 and 11a, but not Rab7 and 14 (Figure 1B). To narrow down the Rab-binding sites in Myo5b, we performed pull down assays using truncated Myo5b tail constructs. While both Rab8a and Rab10 were specifically pulled down with Flag-Myo5b-CC (containing the proximal tail of Myo5b), Rab11a was pulled down with Flag-Myo5b-GTD (Figure 1C). These results are consistent with the previous works that Rab8a and Rab10 bind to the coiled-coil region and Rab11a binds to the GTD of Myo5b [10,16,23–24].

Because the tail of Myo5b, precisely the GTD, is the inhibitory domain, it is possible that the binding of Rab to the tail will interfere the head–GTD interaction of Myo5b, thus affecting the motor activity. To test this possibility, we examined the effects of Rab proteins on the actin-activated ATPase activity (hereafter referred to as ATPase activity) of Myo5b. As shown in Figure 1D, Rab11a substantially enhanced the ATPase activity of Myo5b, whereas all other four Rabs (Rab7, 8a, 10 and 14) had little effect on it. These results indicate that only the Rab that binds to the GTD directly regulates Myo5b motor function. It should be mentioned that no significant ATPase activity was detected in the Rab11a sample alone. Note: The ATPase activity of Myo5b in the absence of Rab11a is lower than our previously reported value [3]. This discrepancy is likely due to different assay conditions, i.e., 0.2 M KCl in the current work and 0.1 M NaCl in the previous one.

### Characterization of Rab11a activation of Myo5b ATPase activity

**Figure 2:**
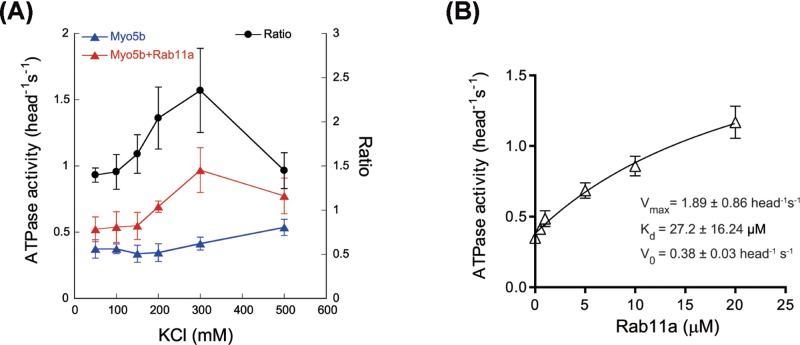
Characterization of Rab11a activation of Myo5b ATPase activity (**A**) Ionic strength dependence of Myo5b-FL ATPase activity in the absence (blue triangle) or presence of 12 μM GST–Rab11a (red triangle). Closed circle, the ratio of Myo5b ATPase activity in the presence of GST–Rab11a versus that in the absence of GST–Rab11a. The ATPase activity was measured as described in Figure 1D, except for the indicated concentrations of KCl. Values are mean ± S.D. from three independent assays. (**B**) Rab11a dependence of Myo5b-FL ATPase activity. The ATPase activity was measured as described above except that 0.2 M KCl and the indicated concentrations of GST–Rab11a were used. The data were fit to a hyperbola *V* = *V*_0_ + *V*_max_ * [Rab11a]/(*K*_d_ + [Rab11a]), where *V*_0_, the ATPase activity in the absence of Rab11a; *V*_max_, the maximal Rab11a-activated ATPase activity; and *K*_d_, the concentration of Rab11a that simulates the ATPase activity to 50% of *V*_max_. Values are mean ± S.D. from three independent assays.

We further characterized the ionic strength-dependence of Rab11a activation of Myo5b ATPase activity. The ratio of Myo5b ATPase activity in the presence of Rab11a versus that in its absence reached the maximum at 200–300 mM KCl (Figure 2A). In the presence of 200 mM KCl, the activation of Myo5b ATPase activity by Rab11a followed the Michaelis–Menten equation, defining the maximal Myo5b ATPase activity in the presence of saturated Rab11a (*V*_max_) of 1.89 ± 0.86 head^−1^s^−1^ and apparent affinity (*K*_m_) of 27.2 ± 16.24 μM Rab11a (Figure 2B). The maximal Myo5b ATPase activity in the presence of saturated Rab11a was approximately 5-fold as that in the absence of Rab11a (the *V*_0_, 0.38± 0.03 head^−1^s^−1^). The relatively low activation (∼2 folds) of Myo5b ATPase activity by 12 μM Rab11a (Figure 1D) is due to the low affinity between Myo5b and Rab11a.

**Figure 3:**
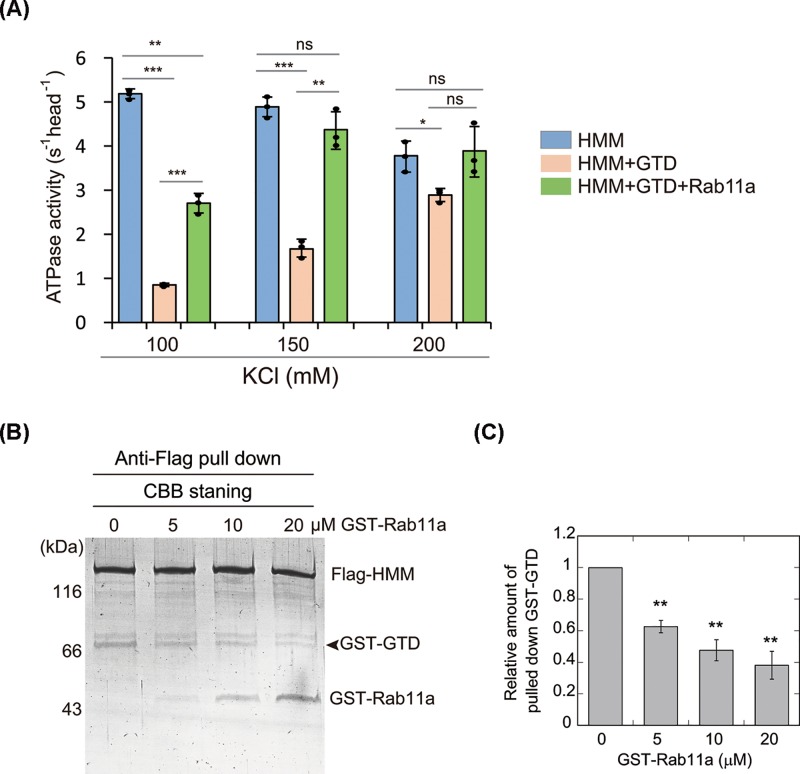
Rab11a activates Myo5b ATPase activity by weakening the head-GTD interaction of Myo5b. (**A**) Rab11a reduces the inhibition of Myo5b–HMM ATPase activity by the GTD. The ATPase activity was measured in the presence of ∼80 nM Myo5b–HMM, 0–5 μM GTD, 0–12 μM GST–Rab11a, 40 μM actin in a solution containing 20 mM MOPS-KOH (pH 7.0), 100–200 mM KCl, 1 mM MgCl_2_, 1 mM DTT, 0.25 mg/ml BSA, 12 μM CaM, 0.5 mM ATP, 2.5 mM PEP, 20 U/ml pyruvate kinase and 1 mM EGTA. Values are mean ± S.D. from three independent assays. ns, no significance; *, *P*<0.05; **, *P*<0.01; ***, *P*<0.001. Panels (**B**) and (**C**) showing that Rab11a weakens the interaction between Myo5b–HMM and Myo5b–GTD. Flag-Myo5b-HMM (0.8 μM), GST-GTD (0.8 μM) and GST–Rab11a (0–20 μM) were pulled down by anti-Flag agarose, and the bound proteins were eluted by Flag peptide. The eluted proteins were subjected to SDS–PAGE and Commassie Blue staining. The remaining of GST–Rab11a in several pull down samples is due to incomplete washing of the beads (for detail, see ‘Materials and methods’ section). (**B**) SDS–PAGE representative of three independent assays. (**C**) The quantification of the pulled down GST–GTD (relative to that in the absence of GST–Rab11a). Values are the mean ± S.D. from three independent assays. ** (*P*<0.01) indicates significant difference compared to the first column.

We also examined the effects of Rab11a on inhibition of Myo5b–HMM ATPase activity by Myo5b–GTD. As shown in Figure 3A, Myo5b–HMM ATPase activity was strongly inhibited by Myo5b–GTD and this inhibition was substantially attenuated by Rab11a. The Rab11a enhancement on the ATPase activity of Myo5b–HMM in the presence of Myo5b–GTD was more pronounced than that on the ATPase activity of Myo5b-FL. This difference can be attributed to the weaker head–GTD interaction in the former (in which the head and the GTD are separated) than that in the later (in which the head and the GTD are covalently connected).

Consistent with the ATPase assay result, anti-Flag pull down assay showed that, in the absence of Rab11a, GST–GTD was stoichiometrically pulled down with Flag-Myo5b-HMM, and GST–Rab11a substantially decreased the amount of GST-Myo5b-GTD pulled down with Myo5b–HMM (Figure 3B). Taken together, above results indicate that Rab11a activates Myo5b ATPase activity by abolishing the head–GTD interaction of Myo5b.

### FIP2 functions as a tether between Rab11a and Myo5b

**Figure 4:**
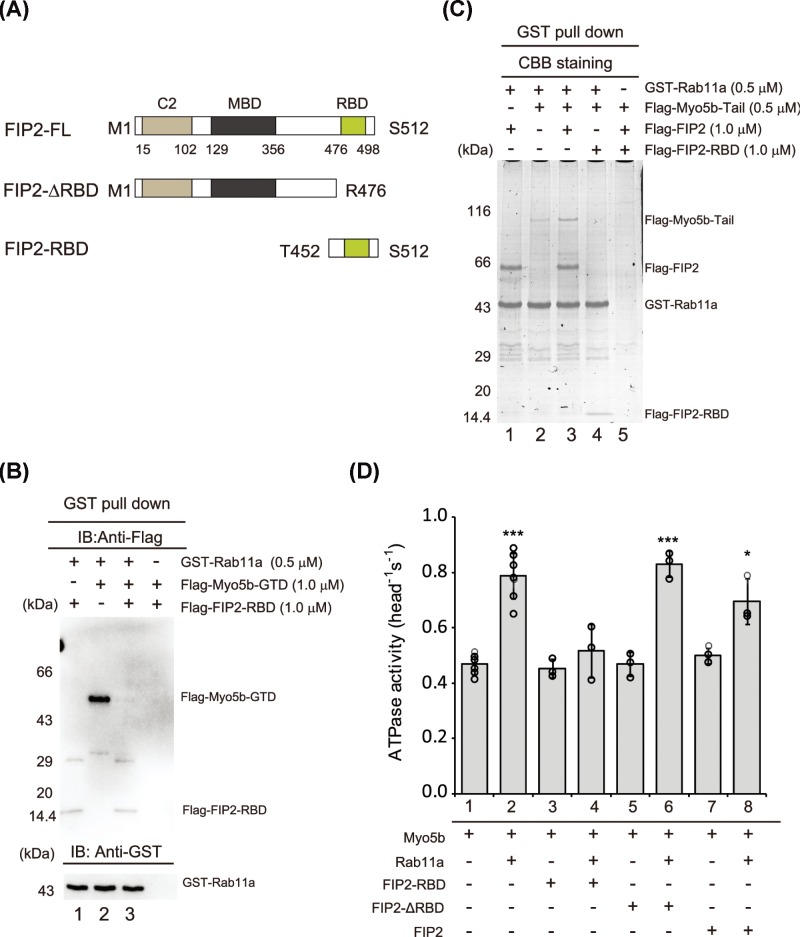
FIP2 bridges Myo5b and Rab11a (**A**) Schematic FIP2 constructs; C2, phospholipid-binding C2 domain; MBD, Myo5-binding domain; RBD, Rab11a-binding domain. (**B**) FIP2–RBD competes with Myo5b–GTD in binding with Rab11a. The interaction between GST–Rab11a and Flag-Myo5b-GTD in the absence or presence of Flag-FIP2-RBD was analyzed by GST pull down assays. The pulled down samples were analyzed by Western blot using Anti-Flag and Anti-GST antibodies. (**C**) FIP2 enhances the interaction between Rab11a–FIP2 and Myo5b–tail. GST pull down of GST–Rab11a with Flag-Myo5b-tail and Flag-FIP2 constructs. The GST pulled down samples were separated by SDS–PAGE and visualized by Coomassie Brilliant Blue Staining. (**D**) Effects of Rab11a and different FIP2 constructs on Myo5b ATPase activity. The ATPase activity of Myo5b was measured as described in Figure 1D, except in the presence of 12 μM indicated proteins (GST–Rab11a and FIP2 proteins). Values are mean ± S.D. from at least three independent assays. * (*P*<0.05) and *** (*P*<0.001) indicate significant differences compared with the first column. The pull down assays (Figure 4B,C) were repeated three times and similar results were obtained.

In addition to Rab11a, FIP2 is also required for the Myo5b-dependent trafficking of recycling endosome [3,25–27]. FIP2 is a multi-domain protein, interacting with Myo5b–tail and Rab11a (Figure 4A). Because Rab11a largely uses the same surface to interact with Myo5b–GTD and FIP2–RBD, it was proposed that FIP2–RBD might compete with Myo5b–GTD in binding to Rab11a [15,16]. To test this possibility, we performed GST pull down of Myo5b–GTD and FIP2–RBD with GST–Rab11a. Both Myo5b–GTD and FIP2–RBD could be individually pulled down with GST–Rab11a (Figure 4B, lanes 1 and 2). However, when both Myo5b–GTD and FIP2–RBD were present, only FIP2–RBD was pulled down with GST–Rab11a (Figure 4B, lane 3). In other words, FIP2–RBD substantially inhibits the interaction between Myo5b–GTD and Rab11a. These results indicate that FIP2–RBD competes with Myo5b–GTD in binding to Rab11a and Rab11a has stronger affinity to FIP2–RBD than to Myo5b–GTD.

Consistent with the pull down results, FIP2–RBD eliminated the Rab11a activation of Myo5b ATPase activity (Figure 4D, column 4), suggesting that substantial amount of Rab11a was segregated by FIP2–RBD. Similar to FIP2–RBD, FIP2 also dampened the Rab11a activation of Myo5b ATPase activity (Figure 4D, column 8). In contrast, deletion of RBD abolishes this effect (Figure 4D, column 6). It should be noted that neither FIP2 nor its truncated constructs alone affect Myo5b ATPase activity (Figure 4D, columns 3, 5 and 7).

In addition to binding to Rab11a, FIP2 directly interacts with Myo5b tail via FIP2–MBD (Figure 4A). We characterized the interactions among Myo5a–tail, Rab11a and FIP2 by GST pull down assay. Substiochiometric amount of FIP2 was pulled down with GST–Rab11a (Figure 4C, lane1), indicating a strong interaction between FIP2 and Rab11a. Interestingly, the amount of Myo5b–tail pulled down with GST–Rab11a was substantially increased by FIP2, but decreased by FIP2–RBD (Figure 4C, lanes 3 and 4). These results indicate that, although FIP2–RBD weakens the direct interaction between Myo5b–GTD and Rab11a, FIP2 substantially enhances the indirect interaction between Rab11a and Myo5b by bridging these two proteins.

## Discussion

### FIP2 and Rab11a collaboratively regulate the tethering and the activation of Myo5b.

Rab11a and its effector protein FIP2 are essential for the Myo5b-dependent transport of recycling endosome. It was proposed that FIP2 functions as an adaptor protein between Myo5b and membrane-bound Rab11a, thus associating Myo5b with vesicle [2,3]. A critical question is how Rab11a and FIP2 assemble Myo5b on the target membrane and trigger the intracellular transport.

Structural analysis shows that Rab11a uses the same surface to interact with Myo5b–GTD and FIP2–RBD [15,16]. Consistently, our pull down assay shows that FIP2–RBD inhibits the interaction between Myo5b–GTD and Rab11a (Figure 4B). Moreover, we show that Rab11a has higher affinity to FIP2–RBD and FIP2 than to Myo5b–GTD (Figure 4B,D), suggesting that Rab11a preferentially binds to FIP2 in the cell.

On the other hand, FIP2 uses two distinct regions to bind to Rab11a and Myo5b tail, thus bridging Rab11a and Myo5b. Yeast-2-hybrid shows that both the GTD and the portion of the proximal tail region (∼200 residues preceding the GTD) of Myo5b are essential for FIP2 binding [2]. We show here that FIP2 alone does not affect Myo5b ATPase activity (Figure 4D), suggesting that the tethering of Myo5b by FIP2 does not activate Myo5b motor function. On the other hand, Rab11a binds to the subdomain-2 of Myo5b–GTD and stimulates Myo5b ATPase activity. These results suggest that the association of Myo5b with membrane is majorly mediated by the FIP2-bridged interaction with Rab11a, rather than by the direct interaction with Rab11a, and the activation of Myo5b motor function is mediated by Rab11a.

**Figure 5:**
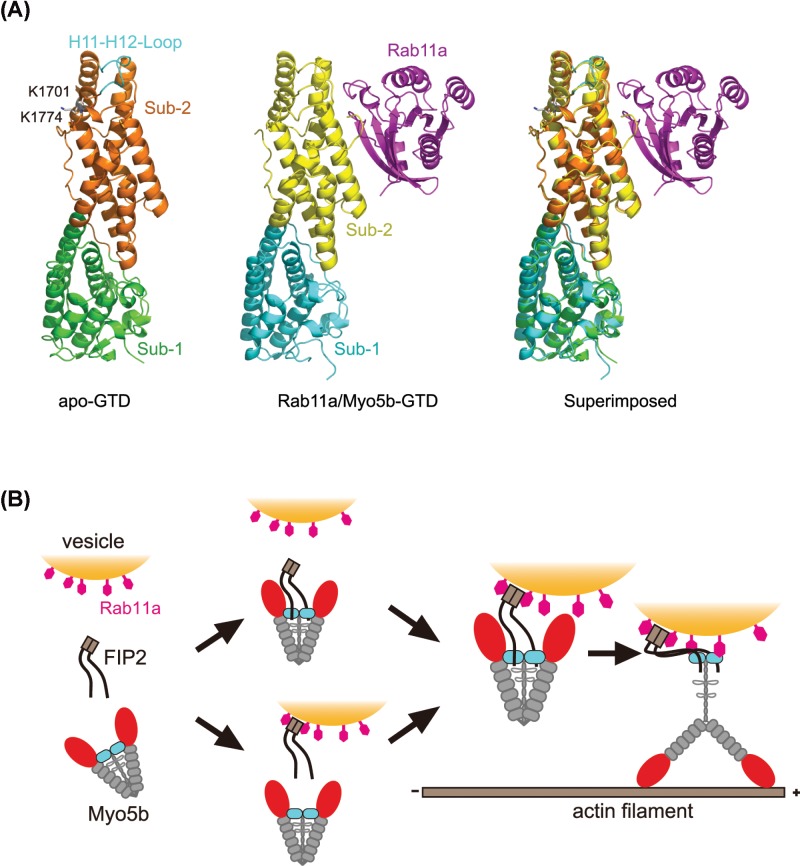
Regulation of Myo5b by Rab11a and FIP2 (**A**) Structural alignment of apo-Myo5b-GTD (PDB ID: 4LNZ) with Myo5b–GTD/Rab11a complex (PDB ID: 4LX0). The two residues (K1701 and K1774) and H11-H12 loop, both critical for the head–GTD interaction of Myo5b, are labeled. (**B**) Proposed model for the association of Myo5b with vesicles via Rab11a/FIP2 and the activation of Myo5b motor function by Rab11a.

Based on our current findings, we propose a dual-regulation mechanism of Myo5b: the association of Myo5b with vesicle and the activation of Myo5b motor function are sequentially regulated by FIP2 and Rab11a as follows (Figure 5B). First, FIP2 bridges the membrane-bound Rab11a and the folded off-state Myo5b. One scenario is FIP2 binds to the membrane-bound Rab11a first, and then recruits the folded off-state Myo5b. Alternatively, FIP2 might interact with Myo5b first, and then bind to the membrane-bound Rab11a. Because FIP2 binding does not affect Myo5b motor function, Myo5b retains the folded off-state. Then, the extra membrane-bound Rab11a binds to the GTD and alleviate the head–GTD interaction, thus inducing the extended on-state Myo5b. Finally, the activated Myo5b hydrolyzes ATP and transport vesicles along actin filament.

According to the dual-regulation mechanism, vesicles might be able to associate with the off-state Myo5b molecules prior to being transported, thus allowing a prompt response to the transportation signals. It is possible that the dual-regulation mechanism is also valid for the regulation of other myosin-5-dependent vesicle transports in cell.

### Activation of Myo5b motor function by Rab11a

By coordinating with different Rabs, Myo5b participates in various intracellular vesicles transport. Rab11a specifically binds to the GTD, whereas Rab8a and Rab10 bind to the proximal tail of Myo5b. We found Rab11a directly regulates Myo5b motor function, whereas Rab8a and Rab10 have no effects on it. This is consistent with the structural and biochemical analysis of Myo5 that the GTD, but not the proximal tail, is the inhibitory domain of Myo5 [18].

A critical question is how the Rab11a binding reliefs the head–GTD interaction, thus activating Myo5b motor function. Several studies suggest that the head-binding site is located in the subdomain-2 of the GTD, comprising the highly conserved basic residues K1701 and K1774 (corresponding to K1706 and K1779 in mouse Myo5a) and the isoform-specific H11–H12 loop (Figure 5A) [21,28]. The Rab11-binding site is located on the surface of the subdomain-2, which is on the side opposite to K1701 and K1774 (Figure 5A). This immediately suggests two possible mechanisms for the activation of Myo5b by Rab11a. One is that the binding of Rab11a to the GTD sterically blocks the head–GTD interaction of Myo5b. Although there is apparently no overlapping between the Rab11a-binding site and the head-binding site in the GTD, the proximity of these two binding sites might prevent the GTD from simultaneous binding of the bulky head domain and Rab11a. Another possibility is that Rab11a allosterically inhibits the head–GTD interaction, as in the case of melanophilin activation of Myo5a [29]. However, superimpose of the structures of Myo5b–GTD with or without Rab11a bound does not reveal any large conformational difference (Figure 5A). Nevertheless, we cannot exclude the possibility that the GTD undergoes a conformational change before or upon binding to the head and this conformational change is inhibited by the Rab11a binding. A high resolution structure of the head–GTD interaction in the folded off-state Myo5 is required to determine the underlined mechanism.

## Abbreviations

CaM: calmodulin
FIP2: the Rab11 family interacting protein 2
GTD: the C-terminal globular tail domain
GTPγS: Guanosine 5’-[γ-thio] triphosphate tetralithium
HMM: heavy meromyosin
Myo5b: myosin-5b
